# Comparison of Bladder Carcinogens in the Urine of E-cigarette Users Versus Non E-cigarette Using Controls

**DOI:** 10.1038/s41598-017-19030-1

**Published:** 2018-01-11

**Authors:** Thomas W. Fuller, Abhinav P. Acharya, Thiagarajan Meyyappan, Michelle Yu, Godugu Bhaskar, Steven R. Little, Tatum V. Tarin

**Affiliations:** 1 The University of Pittsburgh Medical Center, Department of Urology, Pittsburgh, PA USA; 2 The University of Pittsburgh, Department of Chemical and Petroleum Engineering, Pittsburgh, PA USA; 3 The University of Pittsburgh, Department of Chemistry, Pittsburgh, PA USA; 4 The University of Pittsburgh, Department of Bioengineering, University of Pittsburgh, Pittsburgh, PA USA; 5 The University of Pittsburgh, Department of Pharmaceutical Sciences, University of Pittsburgh, Pittsburgh, PA USA; 6 The University of Pittsburgh, Department of Immunology, University of Pittsburgh, Pittsburgh, PA USA; 7 The University of Pittsburgh, Department of Ophthalmology, University of Pittsburgh, Pittsburgh, PA USA; 8 The University of Pittsburgh, McGowan Institute for Regenerative Medicine, University of Pittsburgh, Pittsburgh, PA USA

## Abstract

Electronic cigarette (EC) use is gaining popularity as a substitute for conventional smoking due to the perception and evidence it represents a safer alternative. In contrast to the common perception amongst users that ECs represent no risk initial studies have revealed a complex composition of e-cigarette liquids. Conventional cigarette smoking is a known risk factor for developing bladder cancer and prior reports raise concern some of those causative compounds may exist in EC liquids or vapor. Urine samples were collected from 13 e-cigarette using subjects and 10 non e-cigarette using controls. Five known bladder carcinogens that are either present in conventional cigarettes, products of combustion, or solvents believed to be used in some e-cigarette formulations were quantified by liquid chromatography – mass spectrometry (LC-MS). Analysis of e-cigarette user urine revealed the presence of two carcinogenic compounds, o-toluidine and 2-naphthylamine, at a mean 2.3 and 1.3 fold higher concentration (p-value of 0.0013 and 0.014 respectively). Many of these subjects (9/13) were long term nonsmokers (>12 months). Further study is needed to clarify the safety profile of e-cigarettes and their contribution to the development of bladder cancer given the greater concentration of carcinogenic aromatic amines in the urine of e-cigarette users.

## Introduction

Conventional cigarette smoking is a well-established cause of bladder cancer. The burden of disease is substantial with an estimated 50% of bladder cancers caused by tobacco smoke. In 2013 alone the incidence of new bladder cancers was estimated to be 382,700 with 143,000 deaths. Known carcinogenic urinary compounds from conventional cigarette smoking include aromatic amines, inorganic compounds such as arsenic, polyaromatic hydrocarbons (PAHs), and aldehydes^1–4^.

Electronic cigarettes are gaining popularity as a substitute for conventional cigarette smoking due to the belief and growing evidence that it represents a safer alternative to conventional smoking^5^. This is especially true in youth and young adults where a 900% increase in use amongst high school students has occurred from 2011–2015^6^. In general, these devices are composed of a battery powered heating element that vaporizes a flavored propylene glycol or glycerin based liquid, often containing nicotine, that is inhaled, providing a replacement or adjunct to traditional cigarette use. The lack of combustion associated with these devices and control over the composition of e-cigarette liquids provides the potential for a safer alternative to conventional cigarette smoking. Preliminary studies support the notion that there is a substantially lower amount of carcinogenic aromatic amines in the urine of e-cigarette users than those that use conventional cigarettes^7,8^.

Though safer, initial studies describe that e-cigarette use may not be risk free, as the composition of e-cigarette liquids is complex. E-cigarette liquids may contain, or create by the process of vaporization, known bladder carcinogens including aromatic amines, aldehydes, and PAHs^9^. Frequently, the source of nicotine for e-cigarettes is from the chemical extraction of cured tobacco. The extraction process represents a potential source of tobacco specific compounds in e-cigarettes^6^. More thoroughly defining the risk profile associated with e-cigarette use and bladder cancer is therefore an important public health issue and of substantial interest to urologists and their patients^10,11^.

We compared the urine of non-cigarette smoking e-cigarette users to non-smoking and non-e-cigarette using controls by liquid chromatography-mass spectrometry for known bladder carcinogenic aromatic amines and PAH metabolites to further understand the risk profile of e-cigarette use and bladder cancer. Specifically, we analyzed samples for a noncarcinogenic marker of PAH exposure 1-hydroxypyrene, carcinogenic PAHs including benz(a)anthracene and benzo(a)pyrene, and the carcinogenic aromatic amines o-toluidine and 2-naphthylamine.

## Materials and Methods

### Urine Specimen Collection and Preparation

Urine samples and demographic information, including historical conventional cigarette use and current e-cigarette use patterns, were collected from 13 non-smoking EC using subjects and 10 non-smoking, non-EC using controls following an approval by The University of Pittsburgh Institutional Review Board protocol which included informed consent for participation. The study methods were adherent to the relevant guidelines and regulations from the institutional review board. All subjects were free of conventional cigarette use and other combustible or smokeless tobacco products for at least 6 months by self-report. Five molecules known to be bladder carcinogens or markers of PAH exposure that are either present in conventional cigarettes, common solvents used in some e-cigarette formulations, or produced as a result of vaporization were targeted for analysis. These included benz(a)anthracene, benzo(a)pyrene, 1-hydroxypyrene, o-toluidine and 2-naphthylamine.

Extractions of carcinogens were carried out by a modified version of a previously described protocols^1,2^. Urine samples (5 ml aliquots) were acidified with 1 mL of dilute hydrochloric acid (37%) followed by heating for 1hr at 80 °C. The pH was then adjusted to 6.1–6.4 by adding 10 M NaOH and 3 ml of 2-(N-morpholino)ethanesulfonic acid buffer. The samples were serially extracted with dichloromethane and trace water was removed with sodium sulfate. The extract was heated in the presence of 50 µL pentafluoropropionic anhydride and 25 µL pyridine (to improve MS detection) for 1 hr at 80 °C^12^. Dichloromethane was then added to the samples to make up the volume to 100 µL.

### LC-MS and Quantitation

Prior to subject urine analysis, we ensured accurate detection of the target compounds by resuspending manufacturer standards in dichlormethane and in a pooled control urine specimen followed by LC-MS analysis. 2-napthylamine, o-toluidine, 1-hydroxypyrene, Benz[a]anthracene, Benzo(a)pyrene were purchased and used without further purification (Sigma Aldrich, St. Louis, MO). All other chemicals were of analytical grade or better.

The liquid chromatographic system was a Dionex-Ultimate 3000 (Merge with Thermo Scientific, Bremen, Germany). The column employed was a Hypersil GOLD-C18, 100 × 2.1 mm length and 1.9 μm particle size from Thermo Scientific (USA). The mobile phases were 0.1% formic acid in water (A) and 0.1% formic acid in ACN (B). The gradient program was as follows: 10% B for 0.1 min, then increased linearly to 50% in 8 min, then stay on 50% to 8.5 min and decreased to 10% in 8.6 min, then stay on 10% B to 10 min for equilibration and ready for second run, total run time was 10 min. The flow rate was set constant at 200 μL min^−1^ and injection volume was 5 μL. The LC effluent was pumped on Q-Exactive benchtop Orbitrap-based mass spectrometer (Thermo Scientific, Bremen, Germany) operated in electrospray ionization (ESI) in positive polarity. Nitrogen sheath gas and auxiliary gas flow rate were set at 35 and 20 (arbitrary units), respectively. The capillary temperature was set 250 °C, spray voltage 3.5 kV and the S-lens voltage 60 V. The instrument operated in targeted single ion monitoring (tSIM) by selecting the targeted compounds O-toluidine (m/z 108, [M + H]^+^) and 2-Naphthylamine (m/z 144, [M + H]^+^) at 70,000 resolving power and the AGC target was 1e^6^. We followed the external calibration for quantification purpose and the calibration solutions were prepared in pooled nonsmoker urine sample was spiked at 8 levels for both targeted compounds at 1, 5, 10, 50, 100, 500 and 1000ng/mL. The data was analyzed by using the Xcaliber 2.1 software. The calibration curve was plotted in between the targeted concentrations and the MS area, and it was utilized to determine the concentrations of unknown samples. Student’s two-tailed t-test was performed to determine statistical differences between the non-smoker and the e-cigarette groups using Microsoft Excel 2010. Data including LC-MS protocols and results are available upon request.

## Results

Subjects in the study were predominantly male (69.2%) with a mean age of 39.4 years. All subjects were former smokers with a mean duration of 19.9 years. The mean duration since last cigarette use was 19.9 months. Per self-report, all subjects and controls had abstained completely from conventional cigarettes for at least 6 months prior to specimen collection. Non-smoking, non-e-cigarette using controls had been cigarette, other forms of combustible tobacco product, oral tobacco, and EC free for at least 6 months prior to specimen collection. The average age of the non-smoking, non-e-cigarette using controls was 30.1 (p = 0.06) and 50% male.

Subjects used a variety of e-cigarette products almost entirely with a frequency of >28 times per week (84.6%). EC-using subjects universally reported they believed there were no risks or deleterious health effects to e-cigarette use (Table 1).

**Table 1:** Control and subject demographics, historical conventional cigarette use description, and current e-cigarette use behaviors.

E-cigarette Control & Subject Demographics	
**Control Characteristic (n = 10)**	**Result**
Age	30.1 +/− 7.7
Gender (% male)	50.0%
**E-cigarettes Subject Characteristic (n = 13)**	**Result**
Age	39.4 + /− 13.5
Gender (% male)	69.2%
Duration of conventional cigarette Use (yrs)	19.9 +/− 11.9
Packs per day when smoking	1.3 +/− 0.4
Time since last conventional cigarette use (mo)	19.9 +/− 14.9
Duration of e-cigarette use (mo)	26 +/− 11.3
Frequency of e-cigarette use - % > 28 per week	84.6%
E-cigarette nicotine concentration (mg/ml)	6.8

**Figure 1:**
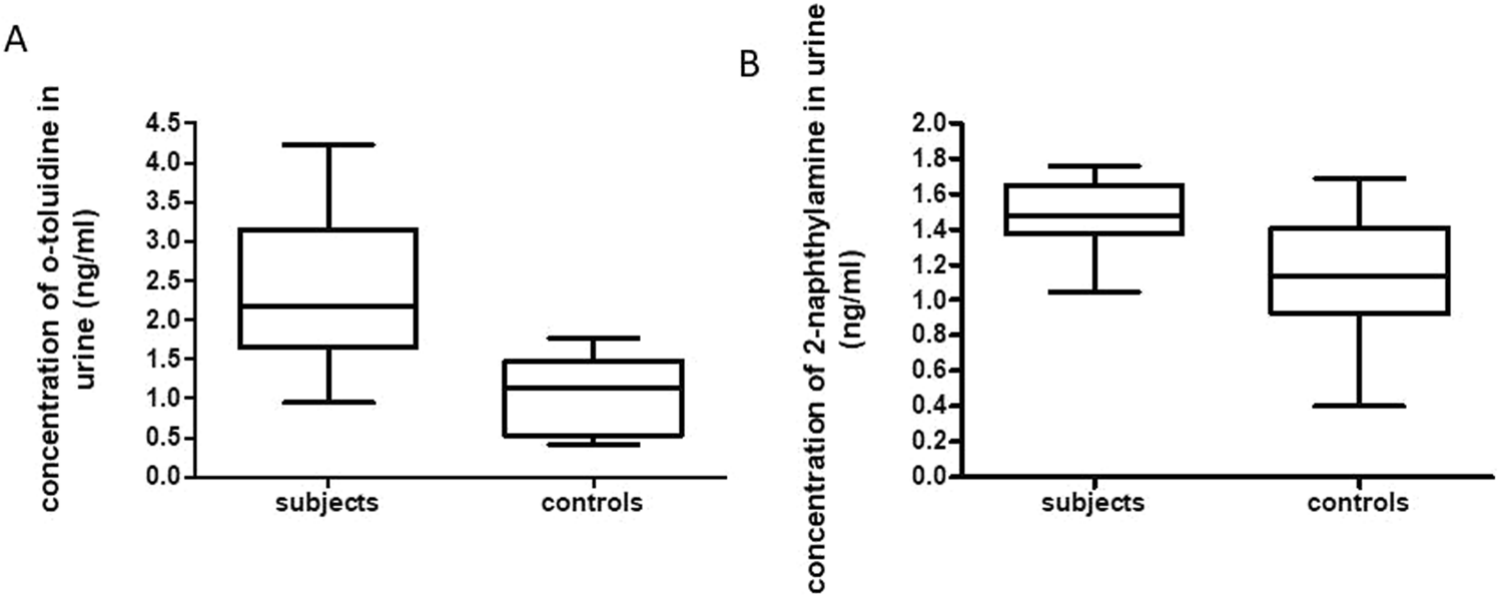
Box plots showing increased levels of (**A**) o-toluidine and (**B**) 2-naphthylamine in the urine samples of e-cigarette users as compared non e-cigarette using controls.

The LC-MS protocol reliably identified standard compounds added to both dichloromethane and a pooled control urine specimen validating the extraction and analysis techniques. The 13 e-cigarette user urines had significantly more of two of the carcinogenic compounds, o-toluidine (p = 0.0013) and 2-naphthylamine (p = 0.014) than the 10 control subjects (N = 9 for o-toluidine as one control had undetectable levels of o-toluidine). The mean concentration of o-toluidine in EC users was 2.33 ng/ml compared to 1.00 ng/ml in non-EC controls. Similarly, the mean concentration of 2-naphthylamine in EC users was 1.46 ng/ml compared to 1.13 in non-EC using controls (Table 2). The tested polyaromatic hydrocarbons were not detectable at our current level of detection and therefore are not included in the table. Figure 1 is a box plot depiction of the distribution of EC user and control aromatic amine concentrations. The other 3 tested urinary carcinogens and markers of PAH exposure were not detected in both the subject and control cohorts within the limits of detection of the extraction method and LC-MS protocol (limit of detection 1 ng/ml in dichloromethane).

**Table 2:** Mean concentration, standard deviation, and range of o-toluidine and 2-naphthylamine in e-cigarettes users and control urine (ng/ml). *N = 9 for o-toluidine controls as one participants o-toluidine level was undetectable.

Urinary Excretion of 2-naphthylamine and o-toluidine		
	EC users	non-EC using controls
**o-toluidine**		
N	13	9*
Mean concentration (ng/ml) +/− SD	2.33 +/− 0.92	1.00 +/− 0.45
Range (ng/ml)	0.94–4.23	0.42–1.67
**2-naphthylamine**		
N	13	10
Mean concentration (ng/ml) +/− SD	1.46 +/− 0.23	1.13 +/− 0.36
Range (ng/ml)	1.05–1.76	0.40–1.69

**Figure 2:**
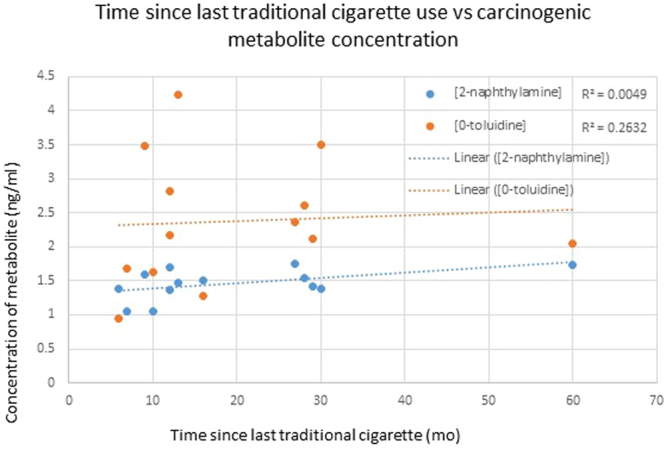
Scatter plot of time since traditional cigarette cessation and concentration of 2-naphthylamine and o-toluidine. There is poor correlation between time since cessation and concentration implying the presence of the carcinogens is not from metabolism of remnant 2-naphthylamine or o-toluidine.

To address the concern that residual carcinogens were present in historically traditional cigarette using controls a Pearson correlation was calculated to compare time since traditional cigarette cessation and carcinogenic metabolite concentration. The Pearson coefficient was 0.51 and 0.07 for 2-naphthylamine and o-toluidine respectively implying there was not a correlation between more recent cigarette use and carcinogenic metabolite concentration (Fig. 2). Coefficients of correlation associating urinary concentration with frequency of e-cigarette use was not possible as nearly the entire subject population reported using e-cigarettes greater than 28 times weekly, which was the highest frequency choice on the multiple-choice questionnaire.

## Discussion

Conventional tobacco smoking is a well described cause of bladder cancer and substantially increases the worldwide burden of this high mortality disease^4^. Electronic cigarettes are gaining popularity as a perceived safe alternative to smoking for both short term smoking cessation efforts and as a long term replacement. The perception of participants in the present study was a unanimous belief that there were no deleterious health effects of e-cigarette use.

Evidence suggests EC are a useful tool for smoking cessation and reduction^13,14^. In addition to smoking cessation efforts, substituting electronic cigarettes for conventional tobacco smoking has a variety of direct immediate benefits including the lack of combustion which decreases the amount of carbon monoxide and carcinogenic PAHs created. If long term use is intended, there is the potential for precise control of e-cigarette liquid composition eliminating carcinogenic compounds and contaminants that have been previously identified in conventional tobacco smoke. Indeed, initial evidence suggests that there are less tobacco-specific nitrosamines in the urine of EC users when compared to conventional cigarette users, substantiating these proposed benefits^7,8,15^.

Despite the clear benefits of electronic cigarettes, their use may not be risk free. A recent published review of studies evaluating EC liquids and aerosols have identified a variety of concerning components, contaminants, and byproducts of vaporization. Aromatic amines, aldehydes, and polyaromatic hydrocarbons are carcinogenic to the human bladder and have been identified as components of e-cigarette liquids^9,15^. Their presence is likely in part due to the lack of historical regulation of electronic nicotine delivery systems (ENDS) and their accompanying e-liquids. Further complicating the use of EC is the variation in usage temperatures between devices and users. While higher temperatures increase the level of nicotine in the vapor, they also increase the levels of carcinogens and toxins in the vapor. Concern regarding the heterogeneity of e-liquid composition in terms of variable nicotine concentrations and carcinogenic constituents lead the Food and Drug Administration (FDA) to extend regulatory authority to include these products in 2016.

The causal role of aromatic amines, including 2-naphthylamine and o-toluidine, in bladder cancer due to traditional cigarette smoke has received significant attention due to their identification as bladder carcinogens from industrial chemical exposure^16–18^. In the present study, o-toluidine and 2-naphthylamine are found in higher concentrations (2.3 and 1.3 fold respectively) in the urine of e-cigarette using subjects than in the urine of non-smoking non-e-cigarette using controls. This is similar to prior studies of traditional cigarette users versus non-smokers where a 2.0 (204 vs. 104 ng/24 h) and 1.9 (20.8 vs. 10.7 ng/24 h) fold higher level of o-toluidine and 2-naphthylamine were found in the smoking population^1^. In contrast, evidence of PAH exposure was not measurable in both the EC users and non-EC using controls in this study limiting the likelihood that EC using participants misrepresented their traditional cigarette use.

The threshold for bladder cancer induction by aromatic amines is unknown, and consequently, acceptable limits of exposure have not been established. This is particularly relevant in genetically susceptible individuals. N-acetylation in the liver can detoxify aromatic amines and is predominantly accomplished through the NAT2 enzyme. The lack of functional NAT2 alleles confers a “slow acetylation” phenotype increasing the risk of bladder cancer when exposed to aromatic amines^19,20^.

The presence of bladder carcinogens in the urine of subjects abstaining from conventional cigarettes at a higher concentration than controls warrants close scrutiny of e-cigarette liquid composition. This is especially true given the unknown limits of exposure to induce bladder cancer in genetically susceptible patients. Current evidence supports that electronic cigarettes are safer alternative to traditional cigarette smoking and some evidence supports their use as a tool for smoking cessation. Therefore, their regulation and restriction must requisitely be balanced by their practical utility.

Limitations to this study include reliance on subject self-reporting of abstinence from conventional cigarette smoking, unknown exposure to second hand smoke, and limited available information on the storage and metabolism of carcinogenic metabolites from past conventional cigarette use. Future larger scale confirmatory studies would benefit from measurement of nicotine or its metabolites, including cotinine, in non e-cigarette, non-smoking controls as an objective verification of their abstinence from nicotine. Nonetheless, from the survey of the participants it was determined that they were not smoking cigarettes. Determining the relationship between bladder carcinogens and nicotine metabolites will be performed in future studies. Additionally, the select compounds analyzed in this study are only a small sampling of the likely contributing carcinogens that are present in conventional tobacco smoke and could be present in e-cigarette liquids or vapors. Lastly, for future studies we intend to expand upon this study for improving our ability to detect any modest differences in other carcinogens between e-cigarettes users and controls.

## Conclusions

E-cigarettes are historically unregulated with a wide variety of formulations. Previous studies have identified bladder carcinogens including benz(a)anthracene and benzo(a)pyrene, aromatic amines, and aldehydes in e-cigarette liquids, vapor, or urine. In our comparison of non-smoking non-e-cigarette using controls versus non-smoking e-cigarette users there are higher levels of bladder carcinogenic 2-naphthylamine and o-toluidine in the urine of e-cigarette users. Most of these subjects last used traditional cigarettes greater than 12 months prior to providing samples for this study. Though evidence supports e-cigarettes are safer overall than traditional cigarettes, the current study suggests e-cigarette use with current variable formulations of liquids and vapor control may not be entirely risk free from bladder cancer perspective.
